# Migraine and Patent Foramen Ovale: Barking up the Wrong Tree?

**DOI:** 10.3389/fneur.2014.00099

**Published:** 2014-06-16

**Authors:** Nicola Morelli, Eugenia Rota

**Affiliations:** ^1^Neurology Unit, Guglielmo da Saliceto Hospital, Piacenza, Italy

**Keywords:** patent foramen ovale, migraine with aura, right-to-left shunt, closure device, white matter lesion

Connection between migraine and patent foramen ovale (PFO) may be defined as a “tower of Babel” or a “vexata quaestio.” Indeed, there is still conflicting evidence as to the causal relationship of PFO in migraine with aura. The PFO is a kind of oblique fissure between the right and the left atrium, formed by the overlap of the septum primum and the septum secundum, guiding right-to-left shunt during fetal life that bypass the lungs. When the baby starts to breathe, the lungs expand and there is a reduction in pulmonary pressure, leading to closure of the shunt in most cases. However, it does not close in about 30% of cases, becoming a potential source of paradoxical embolism (1). Migraine is a disabling condition characterized by recurrent episodes of throbbing headache associated with neuro-vegetative symptoms, including nausea and vomiting, photophobia, and phonophobia, which can also be preceded by transient focal neurological symptoms, like visual or sensory/motor aura. Recently, PFO and its closure have aroused great interest, not only in interventional cardiology, but also in neurology. The frequency of PFO in the population has been reported as 15–35% (1). The life-time prevalence of migraine ranges from 11% in males to 20% in females, with an average of 16% (2). As PFO and migraine are common conditions, their co-occurrence might be coincidental. However, the prevalence of right-to-left shunt has been documented to be significantly (at least twofold) higher in patients suffering from migraine with aura than in healthy controls. This suggests that right-to-left shunt may play a role both in the physiopathology of migraine aura and the increased risk of stroke in migraineurs. PFO accounts for 95% of all right-to-left shunts (3). It has been postulated that a right-to-left shunt may have a causal relationship in migraine attacks with aura. Microembolic paradox phenomena, or serotoninergic metabolites from the venous system, normally inactivated by the pulmonary circulation, may trigger migraine attacks through the induction of cortical spreading depression, owing to the right-to-left shunt (4). The connection between the right-to-left shunt and migraine could also be explained by the presence of transient hypoxic episodes, caused by the blood shunting through the PFO, determining micro-infarctions resulting in migraine attacks (5). However, these hypotheses have many shortcomings and do not explain the PFO-migraine nexus, as reported by Gupta (6) and Sathasivam (7). As a rule, embolic events show an unpredictable hemispheric distribution (6), while migraine pain is typically lateralized, often periodic and predictable, like menstrual migraine. Moreover, unlike PFO, migraine is not manifested at birth. Although migraineurs often have episodes of remission and exacerbation, PFO is not only life-long, but also tends to increase in size with age (1), whilst migraine decreases with aging. These considerations are at odds with a possible link between migraine and PFO. All of which contrary to what one might expect if a PFO was a causal factor in migraine. Indeed, in the presence of a connection one might well expect migraine not to be lateralized, to be evident at birth and to worsen with age. Patients suffering from migraine frequently show white matter lesions on MRI which may be correlated to hypothetical paradoxical micro-embolisms able to lead to micro-infarctions. However, it has been documented that the right-to-left shunt does not increase the burden of white matter lesions (8), again against the migraine/PFO nexus. The percutaneous closure of septal defects with a left-to-right shunt has been associated with improvement of migraine (9). Several studies have been carried out to determine whether closure of the PFO defect is able to affect migraine frequency when the shunting is predominantly right-to-left (10), even if data from epidemiological studies on the relationship between PFO and migraine are controversial. Although non-randomized studies have documented that migraine attack frequency decreases after percutaneous closure of the right to left shunt, to date, the only randomized study (11) carried out on this (migraine intervention with STARFlex^®^ technology) did not confirm these results. Indeed, the primary end-point of cessation of migraine at 6 months post-treatment with percutaneous closure showed no statistically significant difference between the two groups (in intervention and control groups). Migraine/PFO nexus might well represent only the result of a common genetic substratum that, on the one hand, could lead to a predisposition to migraine and, on the other, to endocardiac alteration with persistence of the foramen ovale. A recent report documented that the occurrence of atrial shunts was consistent with autosomal dominant inheritance in some families with aura migraine (12). Even if serendipity does play a role in medicine advances, interpretation may be misleading in the absence of a logically theoretical basis. Despite the theoretical possibility of PFO being at the root of migraine, there is scant basic scientific and clinical evidence to support such a theory. Moreover, the risks involved in PFO closure must not be underestimated. Literature reports that up to 8% of surgical procedures may require transcatheter interventions to manage complications (13). Serious complications, such as the formation of thrombus on the implant device, thromboembolism related to the implant device, cardiac perforation, infective endocarditis, or cardiac arrhythmias have also been reported (13). Therefore, it may be concluded that, currently, PFO closure is not a recommended routine procedure to prevent migraine. Not only is the pathophysiological relevance of this procedure in migraine treatment debatable, but its efficacy has also been inconclusively proven. Moreover, the complications of this procedure may be serious and significant in comparison to the non-life threatening nature of migraine. Further research on PFO in migraine is clearly required before we may consider changing opinion on the aforementioned conclusions. Therefore, RCTs cannot supplant or substitute good clinical sense or justify serendipity. Nevertheless, the speculative links between migraine and PFO continue to intrigue the medical/research community. The best lesson to be learnt from the history of medicine might be that physicians’ enthusiasm should be mitigated by the test of evidence. Let us then strive to improve scientific knowledge before changing clinical practice.

## Conflict of Interest Statement

The authors declare that the research was conducted in the absence of any commercial or financial relationships that could be construed as a potential conflict of interest.
